# Evaluation of Gallic Acid-Coated Gold Nanoparticles as an Anti-Aging Ingredient

**DOI:** 10.3390/ph14111071

**Published:** 2021-10-22

**Authors:** Yun-Zhen Wu, Yen-Yu Tsai, Long-Sen Chang, Ying-Jung Chen

**Affiliations:** 1 Department of Fragrance and Cosmetic Science, Kaohsiung Medical University, Kaohsiung 807, Taiwan; m06m060322@gmail.com (Y.-Z.W.); yenn755@gmail.com (Y.-Y.T.); 2 Institute of Biomedical Sciences, National Sun Yat-Sen University, Kaohsiung 804, Taiwan; lschang@mail.nsysu.edu.tw; 3 Drug Development and Value Creation Research Center, Kaohsiung Medical University, Kaohsiung 807, Taiwan; 4 Department of Medical Research, Kaohsiung Medical University Hospital, Kaohsiung 807, Taiwan

**Keywords:** gold nanoparticle, gallic acid, matrix metalloproteinase-1, anti-aging, high glucose

## Abstract

Hyperglycemic environment-induced oxidative stress-mediated matrix metalloproteinase-1 (MMP-1) plays a crucial role in the degradation of the extracellular matrix (ECM), which might contribute to premature skin aging. Synthesized, environmentally friendly gallic acid-coated gold nanoparticles (GA–AuNPs) have been evaluated as an anti-aging antioxidant. Their microstructure was characterized by transmission electron microscopy (TEM), which showed that GA–AuNPs are spherical when prepared at pH 11. Dynamic light scattering (DLS) analysis revealed that the average hydrodynamic diameter of a GA–AuNP is approximately 40 nm and with a zeta potential of −49.63 ± 2.11 mV. Additionally, the present data showed that GA–AuNPs have a superior ability to inhibit high glucose-mediated MMP-1-elicited type I collagen degradation in dermal fibroblast cells. Collectively, our data indicated that high-glucose-mediated ROS production was reduced upon cell treatment with GA–AuNPs, which blocked p38 MAPK/ERK-mediated c-Jun, c-Fos, ATF-2 phosphorylation, and the phosphorylation of NFκB, leading to the down-regulation of MMP-1 mRNA and protein expression in high glucose-treated cells. Our findings suggest that GA-AuNPs have a superior ability to inhibit high-glucose-mediated MMP-1-elicited ECM degradation, which highlights its potential as an anti-aging ingredient.

## 1. Introduction

Skin aging can generally be attributed to extrinsic or intrinsic factors. Intrinsic aging refers to the effects of hormonal, genetic, physiological, and pathological changes over time. Extrinsic factors concern changes in environmental, lifestyle, diet, exposure to sunlight, smoke and pollution. Solar radiation is the most important extrinsic factor associated with premature skin aging [1]. Another is a high serum glucose level [2]. Diabetic patients often have skin that appears aged because the collagen in the dermal connective tissue is fragmented [3]. Exposing dermal fibroblasts and skin tissue to a hyperglycemic environment causes premature cellular senescence and induces the production of matrix metalloproteinase-1 (MMP-1) [2,4], the major protease that causes collagen fragmentation. High glucose exposure also leads to increased levels of intracellular reactive oxygen species (ROS) in several different cell types [5,6,7,8]. It has been shown that ROS stimulate signal transduction, resulting in the increased expression of MMP-1 [9]. The antioxidants defend against ROS by various mechanisms, including (1) scavenge free radicals; (2) maintain the endogenous antioxidant-protective system; (3) inhibit free radical generating enzyme activity [10,11]. Phenolic acids can act as free radical scavengers either by donating a hydrogen atom or acting as electron donors [12,13]. Phytochemicals, including (-)-epigallocatechin-3-O-gallate (EGCG), resveratrol, ferulic acid, and protocatechuic acid show potential as anti-skin-aging treatments for their antioxidant properties and inhibition of MMP-1 in in vitro assays and for anti-wrinkle properties as shown in in vivo skin tests [14,15,16]. Strategies for reducing the generation of intracellular ROS have been developed to prevent MMP-1 upregulation and protect skin from aging.

**Scheme 1:**
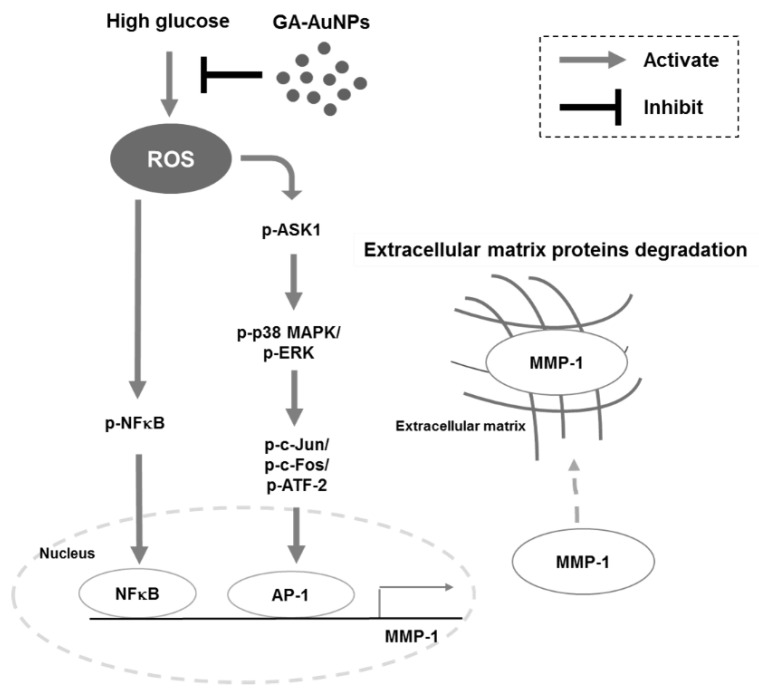
The pathways elucidate the suppressive effect of GA–AuNPs on high glucose-induced MMP-1 upregulation in CCD-966SK cells. Treatment with high glucose induced ROS generation and activation of NFκB and AKS1-p38 MAPK/ERK-mediated AP-1 (c-Jun, c-Fos, ATF-2), leading to MMP-1 upregulation and increased MMP-1-mediated collagen degradation. GA–AuNPs inhibited ROS production, and phosphorylation of AKS1/MAPKs/AP-1 and NFκB in high glucose-treated cells. Consequently, GA–AuNPs terminated high glucose-induced MMP-1 expression and inhibited MMP-1-meditated collagen degradation in the extracellular matrix.

Recent studies revealed gold nanoparticles as a potential antioxidant agent having low cytotoxicity and good cell permeability [17]. Compared with gold salts, biologically produced gold nanoparticles spread more effectively to scavenge free radicals [18]. Antioxidant AuNPs are mostly derived from plant extracts, and from these many phenolic acids have been investigated as either reducers or stabilizers for metal nanoparticle synthesis [19]. The phenol functional group has an absorbent ability on metal surfaces [19]. Gallic acid is a phenolic compound that exhibited antihyperglycemic, anti-lipid peroxidative, and antioxidant effects on streptozotocin-induced diabetic rats [20]. Pang et al. reported that gallic acid suppressed MMP-1 protein expression in human nasopharyngeal carcinoma cells [21]. Furthermore, gallic acid has been reported to inhibit ultraviolet (UV) radiation-induced ROS and MMP-1 activity, indicating that it is a candidate for preventing skin photoaging [22]. In our previous studies, compared with gallic acid, gallic acid-coated gold nanoparticles (GA–AuNPs) exhibited a superior ability to suppress epidermal growth factor (EGF)-mediated MMP-9 expression in breast cancer cells, suggesting that AuNPs could serve as a vehicle to enhance the functional activity of gallic acid [23]. It is noteworthy that the anti-aging activity of GA–AuNPs has not yet been studied. To evaluate the anti-aging activity of GA–AuNPs on human dermal fibroblasts, we tested the effect of GA–AuNPs on MMP-1 expression in the high glucose-treated dermal fibroblast cell line CCD-966SK. Our results highlight the inhibitory pathway of high glucose-mediated ROS through GA-AuNPs, which further reduced MMP-1 expression (Scheme 1).

## 2. Results

### 2.1. Characterization of Synthesized GA–AuNPs

**Figure 1:**
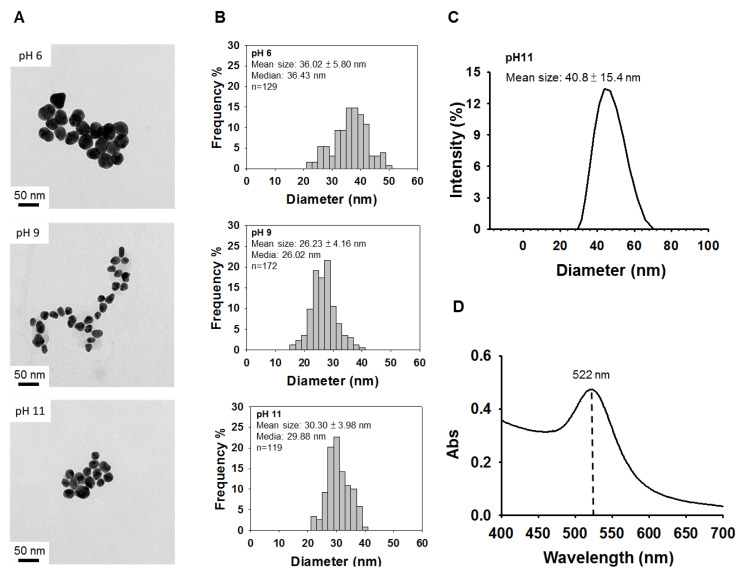
Characterization of gallic acid-coated gold nanoparticles (GA–AuNPs) synthesis. (**A**) TEM images for GA–AuNPs prepared at pH 6, 9, and 11. (**B**) TEM-derived size distributions of GA-AuNPs. (**C**) The size distribution of GA–AuNPs prepared at pH 11 by DLS measurement. (**D**) UV–visible absorption spectra for GA–AuNPs prepared at pH 11.

The effects of pH during the synthesis of nanoparticles (NPs) on size distribution and shape variation were reported by Patungwasa et al. [24]. Figure 1A shows the transmission electron microscopy (TEM) image of GA–AuNPs prepared at different pH levels (respectively 6, 9, and 11). The median diameter, average diameter, and standard deviations of GA–AuNPs are presented in Table 1, which were taken from the results of TEM image analyses (Figure 1A, Supplementary Figure S1). The images reveal that GA–AuNPs prepared at pH 6 and 9 showed irregular shapes. According to TEM analysis, GA–AuNPs had spherical shapes with an average diameter of 30.30 ± 3.98 nm when the synthesis was conducted at pH 11 (Figure 1B). The average hydrodynamic diameter of the GA–AuNPs (pH 11) was 40.8 ± 15.4 nm using dynamic light scattering (DLS) measurement (Figure 1C). The average diameter of the GA–AuNPs from DLS was slightly larger than that of TEM. This may have been the result of interference with the dispersant in the hydrodynamic diameter and the partial agglomeration and aggregation of NPs during DLS measurement [25]. Figure 1D presents the absorption spectra of the suspension of synthesized GA–AuNPs (pH 11), showing the typical curve with the absorption maximum at 522 nm associated with the surface plasmon resonance bond [26]. The cationic GA–AuNPs depolarized the membrane, which resulted in the breaking of cell membranes. In contrast, the negatively charged gold nanoparticles were much safer [27]. Thus, we aimed to prepare GA–AuNPs with a negative surface charge. A zeta potential of 61.30 ± 2.84 mV, 48.26 ± 1.84 mV, and −49.63 ± 2.11 mV of the GA–AuNPs was prepared by pH 6, pH 9, and pH11, respectively (Table 2). Thus, the gold nanoparticle reduced by gallic acid prepared at pH 11 were used in the subsequent experiments.

### 2.2. GA–AuNPs Prevents High Glucose-Induced MMP-1 Expression in Human Dermal Fibroblasts

**Figure 2:**
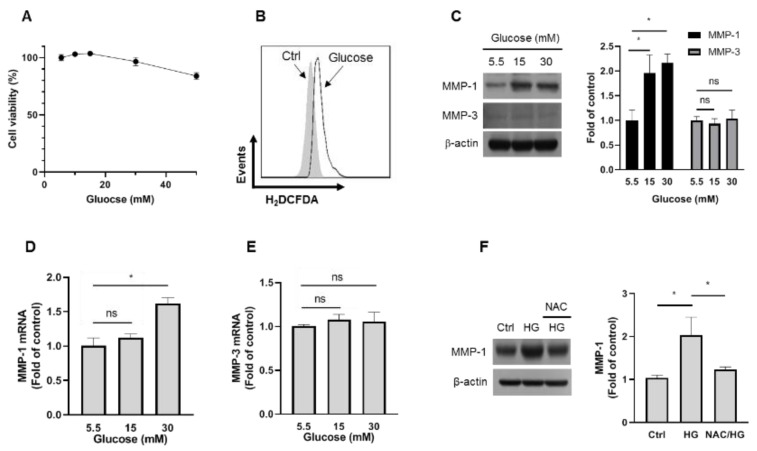
High glucose-upregulated MMP-1 and ROS expression in human dermal fibroblasts. (**A**) Effect of glucose on the cell viability of CCD-966SK cells. Cells were incubated with different concentrations of glucose for 24 h. The results are expressed as the percentage of cell viability relative to the control. (**B**) High glucose induced an increase in ROS generation. CCD-966SK cells were incubated with 30 mM glucose for 24 h. Cellular ROS content was measured by a flow cytometer in an H_2_DCFDA fluorogenic probe assay. (**C**–**E**) CCD-966SK cells were treated with 5.5, 15, or 30 mM of glucose for 24 h. (**C**) Effect of glucose on MMP-1 and MMP-3 protein expression in human dermal fibroblasts. (**Left panel**) Western blot analyses. (**Right panel**) Quantification of MMP-1, MMP-3 expression from Western blot analyses (* *p* < 0.05; NS, statistically insignificant). Effect of glucose on transcription levels of MMP-1 (**D**) and MMP-3 (**E**). The MMP-1 and MMP-3 mRNA levels were analyzed by real-time RT-PCR (* *p* < 0.05; NS, statistically insignificant). (**F**) Effect of N-acetylcysteine (NAC) on high glucose-induced MMP-1 protein expression. CCD-966SK cells were pretreated with or without 5 mM NAC for 1 h and incubated with 30 mM glucose for 24 h. (**Left panel**) Western blot analyses. (**Right panel**) Quantification of MMP-1 expression from Western blot analyses (* *p* < 0.05).

ROS is one of the major factors that play a key role in skin aging [28]. A previous study revealed that high glucose induced an increase in ROS generation in dermal fibroblasts [8]. As shown in Figure 2A, treatment of cells with glucose ranging from 5.5 to 50 mM showed an approximately 15–20% decrease in viability. Results of cytometric flow analysis revealed a significant increase in ROS generation in high glucose treatment groups compared to the control group (Figure 2B). Figure 2C shows that high glucose increased MMP-1 but not MMP-3 protein expression. Real-time RT-PCR analyses revealed that the MMP-1 mRNA level in high glucose-treated CCD-966SK cells was higher than in untreated control cells, while high glucose treatment did not affect the MMP-3 mRNA level (Figure 2D,E). Treatment with N-acetylcysteine (NAC, ROS scavenger) countered the effect of high glucose on MMP-1 expression, indicating ROS pathway involvement in high glucose-induced MMP-1 upregulation (Figure 2F). These results suggested that glucose-elicited ROS increase was involved in MMP-1 expression in glucose-treated cells.

**Figure 3:**
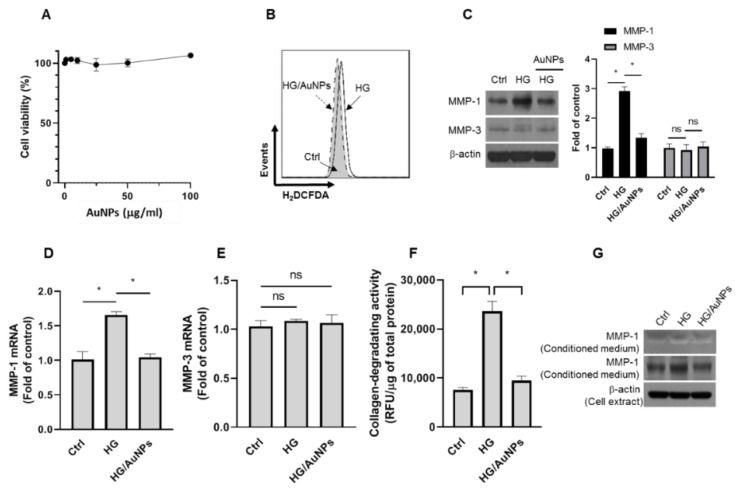
Effect of GA–AuNPs on MMP-1 expression in high glucose-treated CCD-966SK cells. (**A**) Effect of GA–AuNPs on the viability of cells. CCD-966SK cells were treated with GA–AuNPs (0, 1, 5, 10, 25, 50, or 100 μg/mL) for 24 h. The results were expressed as the percentage of cell viability relative to the control. (**B**–**G**) CCD-966SK cells were pretreated with 10 μg/mL GA–AuNPs for 1 h, and then incubated with 30 mM glucose for 24 h. (**B**) Effect of GA–AuNPs on ROS generation in high glucose-treated cells. Cellular ROS content was measured by flow cytometer in an H_2_DCFDA fluorogenic probe assay. (**C**) Effect of GA–AuNPs on MMP-1 and MMP-3 protein expression in high glucose-treated cells. (**Left panel**) Western blot analyses; (**right panel**) quantification of protein expression from Western blot analyses (* *p* < 0.05). Effect of GA–AuNPs on transcription levels of MMP-1 (**D**) and MMP-3 (**E**) mRNA in high glucose-treated cells. The MMP-1 and MMP-3 mRNA levels were analyzed by real-time RT-PCR (* *p* < 0.05; NS, statistically insignificant). (**F**) Effect of GA–AuNPs on collagen-degrading activity on a culture medium of high glucose-treated cells. Collagen-degrading activity was analyzed by a fluorescence microplate reader at Ex/Em = 490/520 nm (* *p* < 0.05; NS, statistically insignificant). (**G**) Effect of GA–AuNPs on MMP-1 in culture medium of high glucose-treated cells. Collagen zymography and Western blot detecting MMP-1 from conditioned medium. Western blot detecting β-actin from cell extracts.

To examine the cell viability of GA–AuNPs in CCD-966SK cells, the cells were exposed to different doses of GA–AuNPs (1, 5, 10, 25, 50, and 100 μg/mL) and then cultured for 24 h. The results showed that GA–AuNPs did not cause notable cell toxicity (Figure 3A). The increase in high glucose-induced ROS on dermal fibroblasts was inhibited by treatment with 10 μg/mL GA–AuNPs (Figure 3B). As shown in Figure 3C, 10 μg/mL GA–AuNPs reduced high glucose-stimulated MMP-1 protein expression, but did not affect MMP-3. Real-time RT-PCR analyses showed that treatment with GA–AuNPs arrested the high glucose-elicited increase in MMP-1 mRNA levels (Figure 3D); however, levels of MMP-3 mRNA were insignificantly affected (Figure 3E). MMP-1, a collagenase, was assayed by a collagen-degrading activity assay and collagen zymography. Its collagen-degrading activity significantly increased in the high glucose-treated cells, while treatment with GA–AuNPs inhibited activity in CCD-966SK (Figure 3F). Collagen zymography, an electrophoretic method for measuring proteolytic activity, demonstrated that high glucose-induced cleavage of type I collagen resulted in the appearance of a 54 kDa band, which Western blot analysis confirmed to be MMP-1 (Figure 3G). This figure also showed that GA–AuNPs attenuated the secretion of MMP-1 from human dermal fibroblasts CCD-966SK into the culture medium in a high glucose stimulation. The present study demonstrated that high glucose-induced MMP-1-mediated type I collagen degradation can be inhibited by treatment with GA–AuNPs. 

### 2.3. GA–AuNPs Inhibited MMP-1 Expression in Glucose-Induced Fibroblast Cells through ASK1, MAPKs, NFκB and AP-1 Pathways

**Figure 4:**
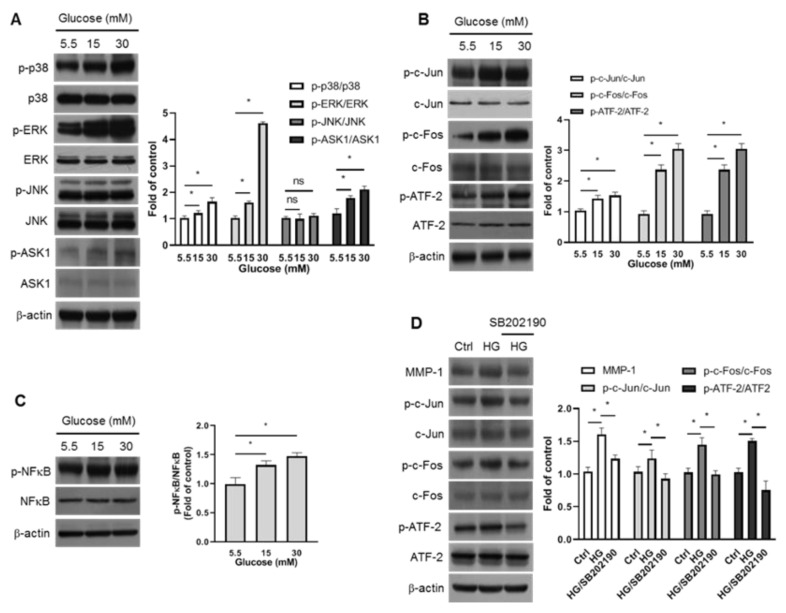

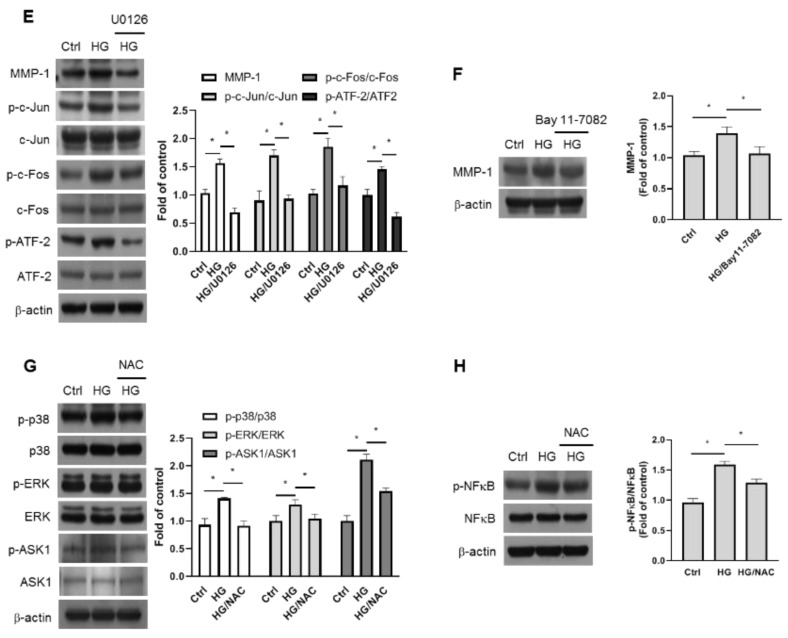
Suppression of p38 MAPK/ERK-mediated c-Jun/c-Fos/ATF-2 phosphorylation and NFκB phosphorylation downregulated MMP-1 expression in high glucose-treated cells. (**A**–**C**) CCD-966SK cells treated with 5.5, 15, or 30 mM of glucose for 24 h. (**Left panel**) Western blot analyses. (**Right panel**) Quantification of protein expression from Western blot analyses (* *p* < 0.05; NS, statistically insignificant). (**A**) Western blot analyses of phosphorylated MAPKs and phosphorylated ASK1 in glucose-treated cells. (**B**) Western blot analyses of phospho-c-Jun, phospho-c-Fos, and phospho-ATF-2 in glucose-treated cells. (**C**) Western blot analyses of phospho-NFκB in glucose-treated cells. (**D**–**H**) CCD-966SK cells were pretreated with 10 μM SB202190 or U0126 or 5 μM Bay11-7082 or 5 mM NAC for 1 h and then incubated with 30 mM glucose for 24 h. (**Left panel**) Western blot analyses. (**Right panel**) Quantification of protein expression from Western blot analyses (* *p* < 0.05; NS, statistically insignificant). Effect of SB202190 (**D**) or U0126 (**E**) on MMP-1, phospho-c-Jun, phospho-c-Fos, and phospho-ATF-2 protein expression in high glucose-treated cells. (**F**) Effect of Bay11-7082 on MMP-1 protein expression in high glucose-treated cells. (**G**) Effect of NAC on the levels of phosphorylated MPAKs and phosphorylated ASK1 in high glucose-treated cells. (**H**) Effect of NAC on the levels of phospho-NFκB in high glucose-treated cells.

The serine/threonine kinase ASK1 (Apoptosis signal-regulating kinase) belongs to the mitogen-activated protein kinase (MAPK) family that is activated by ROS [29]. The results showed that increased levels of phospho-p38 MAPK, phospho-ERK, and phospho-ASK1 were induced by glucose compared to the control (Figure 4A). Given that AP-1 and NFκB are said to be involved in regulating MMP-1 expression in dermal fibroblasts [30,31,32], glucose was seen to induce a significant increase in the phosphorylation of AP-1 (c-Jun, c-Fos, ATF-2) and NFκB compared to the control as represented in Figure 4B,C. Treatment with SB202190 (p38 MAPK inhibitor) or U0126 (MEK1 and MEK2 inhibitor) suppressed the high glucose-induced MMP-1 upregulation, and the activation of c-Jun, c-Fos, and ATF-2 (Figure 4D,E), reflecting that activated p38 MAPK and ERK supported c-Jun, c-Fos, and ATF-2 phosphorylation. Moreover, Bay11-7082 (IKK inhibitor) inhibited the glucose-induced MMP-1 protein upregulation (Figure 4F), indicating that NFκB is involved in MMP-1 expression. These results emphasized the notion that p38 MAPK/ERK-mediated c-Jun/c-Fos/ATF-2 activation and NFκB activation were involved in regulating high glucose-induced MMP-1 upregulation. NAC pretreatment prevented high glucose from increasing p38 MAPK, ERK, ASK1 (Figure 4G), and NFκB phosphorylation (Figure 4H), suggesting that ROS are located at the upstream position for regulating p38 MAPK, ERK, ASK1, and NFκB activation in high glucose-treated cells. These results indicated that ROS-mediated p38 MAPK/ERK/AP-1 and NFκB pathways are responsible for high glucose-stimulated MMP-1 expression in CCD-966SK cells.

**Figure 5:**
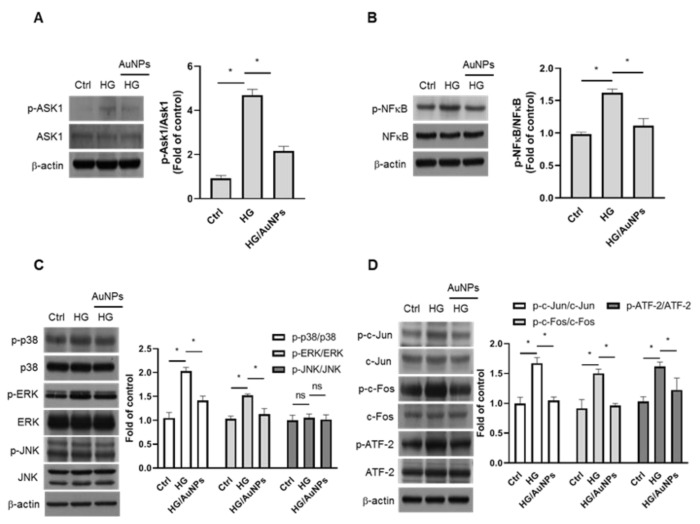
Effect of GA–AuNPs on levels of phosphorylated ASK1 (**A**), NFκB (**B**), MAPKs (**C**), and c-Jun/c-Fos/ATF-2 (**D**) in high glucose-treated CCD-966SK cells. The cells were pretreated with 10 μg/mL for 1 h, and then incubated with 30 mM glucose for 24 h. (**Left panel**) Western blot analyses. (**Right panel**) Quantification of protein expression from Western blot analyses (* *p* < 0.05; NS, statistically insignificant).

Figure 5A shows that treatment with GA–AuNPs inhibited high glucose-induced upregulation of AKS1 phosphorylation. These results indicate that GA–AuNPs block high glucose-induced MMP-1 signaling via the downregulation of ROS-mediated ASK1 signaling pathways. To confirm that the inhibitory activity of GA–AuNPs on NFκB and p38 MAPK/ERK/AP-1 pathways was crucial for downregulating MMP-1 expression in high glucose-treated cells, an analysis of the effect of GA–AuNPs on NFκB, MAPKs, and AP-1 was conducted. The results showed that GA–AuNPs significantly reduced NFκB in high glucose cells (Figure 5B). Figure 5C shows that GA–AuNPs treatment reduced high glucose-induced p38 MAPK and ERK phosphorylation. It also markedly downregulated phospho-c-Jun, phospho-c-Fos, and phospho-ATF-2 in high glucose CCD-966SK cells (Figure 5D). These results suggested that decreased NFκB and AP-1 activation by GA–AuNPs led to reduced MMP-1 expression in high glucose-treated cells.

## 3. Discussion

In this work, we reported that high levels of glucose induced the upregulation of MMP-1, and that GA–AuNPs, synthetized by a green method using gallic acid inhibited the upregulation of MMP-1. AuNPs have been used extensively in skin products such as anti-aging creams [33]. The methods used to synthesize AuNPs from naturally occurring reagents are environmentally friendly, safe, and simple [34]. Gallic acid, used as the reducing and capping agent, has been shown to suppress the upregulation of UVB-induced MMP-1 expression [22]. MMP-1 is the major collagenase capable of destroying type I and III collagen thereby degrading connective tissue resulting in photoaging [35]. Our data showed that dermal fibroblasts cultured in 30 mM D-glucose caused an almost two-fold increased MMP-1 protein expression compared to cells cultured in low-glucose (5.5 mM). Fibroblasts from healthy individuals cultured in high glucose (23–30 mM) increased MMP-1 expression compared to cells cultured in low glucose conditions [8,36]. Argyropoulos et al. reported that MMP-1 is significantly elevated in diabetic human skin, which leads to chronic alterations in the collagenous dermal extracellular matrix (ECM) [37]. The accumulation of fragmented collagen fibrils caused by MMP-1 impaired the integrity and function of the skin structure, thus accelerating aging and age-related disorders such as delayed wound healing [37,38]. Notably, our data revealed that GA–AuNPs effectively attenuated the high glucose-mediated upregulation of MMP-1 in dermal fibroblasts, suggesting that they are a potential treatment for high glucose-induced disruption of ECM homeostasis.

MMP-1 gene transcription is regulated by the activator protein AP-1, which consists of c-Jun, c-Fos, and ATF-2, a well-known MAPK-regulated signaling pathway [39]. Nevertheless, NFκB activation plays a key role in cytokine-induced MMP-1 expression [32,40]. Our data showed that glucose-induced ROS generation elicits ASK1/p38 MAPK/ERK and NFκB activation in human dermal fibroblasts, also known as the CCD-966SK cell line. Our data revealed that the suppression of p38 MAPK/ERK-mediated c-Jun/c-Fos/ATF-2 phosphorylation attenuated glucose-induced MMP-1 upregulation. Moreover, treatment with Bay11-7082 (IκBα inhibitor) modestly reduced glucose-induced MMP-1 upregulation, indicating that the NFκB pathway was related to MMP-1 expression. The data in the present study showed that both AP-1 and NFκB transcriptional factors are involved in high glucose-induced MMP-1 upregulation. Recent studies noted that interaction of c-Jun or c-Fos with NFκB occurs on many gene regulatory regions, and that transcription factor complexes assist with DNA binding in chromatin and synergistically enhance their targeted gene expression [41]. Our study revealed that activated NFκB and AP-1 can be bridged to form complexes that facilitate the formation of DNA looping in MMP-9 promoter and thus MMP-9 expression [42,43]. These observations may explain the role of both NFκB and AP-1 in regulating high glucose-elicited MMP-1 upregulation. Furthermore, our data suggested that GA–AuNPs suppress high glucose-mediated ROS generation, leading to the inhibition of ASK1/MAPKs/AP-1 and NFκB. As a result, GA–AuNPs inhibit high glucose-induced AP-1/NFκB-mediated MMP-1 upregulation.

The development of gallic capped-gold nanoparticles was done in hopes of enhancing the performance and bioavailability of the active ingredients in cosmetics products. It was reported that AuNPs particles ≤200 nm could penetrate the stratum corneum (SC) [44]. The SC is the principle barrier for the human skin, and the transdermal penetration of AuNPs is done via intercellular, transcellular and hair follicular pathways [45,46]. The data indicated that the size of the gallic acid-coated gold nanoparticles was ~40 nm, and Vogt et al. showed that these were able to penetrate the perifollicular dermis via the hair follicle [47]. The shape of the AuNPs depended on growth conditions such as pH value, additives, and temperature [48]. Spherical AuNPs were obtained by increasing seed particle concentration and eliminating anisotropic growth [48]. The deprotonation of gallic acid at pH 11 could possibly lower the nucleation energy barrier of AuNPs, leading to increased seed particle concentration. Therefore, the spherical shape of GA–AuNPs was obtained.

The zeta potential is related to the effective surface charge on a particle in solution. Gallic acid molecules contain an aromatic ring, a carboxylic group, and three hydroxyl groups, which together have four potential acidic protons with pKa values of 4.0 (carboxylic acid), and 8.7, 11.4, and >13 (phenolic OHs) [49]. The GA–AuNPs, prepared at pH 11, exhibited a negative zeta potential, indicating an increase in particle binding properties due to deprotonation in both the carboxylate group and hydroxyl group [50]. In addition, a significant increase in the absolute value of the zeta potential was acknowledged when GA–AuNP was prepared at pH 11. This result indicated that the adsorbed deprotonation of gallic acid improves stabilization and prevents GA–AuNP agglomeration. The variation in the polydispersity index values was consistent with the zeta potential results. In general, the NPs were considered as monodisperse when the polydispersity index was less than 0.2 [51]. The sample synthesized at pH 11 had a lower polydispersity index (0.141), revealing well-dispersed GA–AuNPs.

## 4. Materials and Methods

### 4.1. Reagents and Antibodies

Gallic acid, hydrogen tetrachloroaurate (HAuCl_4_), and collagen I were obtained from Sigma-Aldrich Inc. (St. Louis, MO, USA). The CellTiter 96^®^ aqueous one-solution proliferation assay kit was obtained from Promega Corporation Inc. (Madison, WI, USA). H_2_DCFDA (2′,7′-dichlorodihydrofluorescein diacetate) was obtained from Thermo Fisher Scientific (Waltham, MA, USA). Anti-MMP-1 and anti-MMP-3 were purchased from Abcam (San Francisco, CA, USA). Anti-β-actin and peroxidase conjugated secondary antibodies were obtained from EMD Millipore Inc. (Burlington, MA, USA). Anti-phospho-NFκB p65 (Ser536), anti-NFκB p65, anti-phospho-ERK, anti-ERK, anti-phospho-JNK, anti-JNK, anti-phospho-p38 MAPK, anti-p38 MAPK, anti-phospho-ASK1, anti-ASK1, anti-phospho-c-Jun, anti-c-Jun, anti-phospho-c-Fos, anti-c-Fos, anti-phospho-ATF-2, and anti-ATF-2 antibodies were the products of Cell Signaling Technology (Beverly, MA, USA). Cell culture supplies were purchased from GIBCO/Life Technologies Inc. (Carlsbad, CA, USA).

### 4.2. Synthesis of GA–AuNPs

The reduction method to prepare GA–AuNPs used HAuCl_4_ with a slight modification [19]. The mixture was prepared by trickling gallic acid (10 mM, 0.1 mL, 50 °C) into 10 mL HAuCl_4_ (2.5 mM, pH 6, 9 or 11) solution, then stirred for 6 h at room temperature. H7100 transmission electron microscopy (TEM) and energy-dispersive detector (EDS) (Hitachi High-Technologies Co., Tokyo, Japan) were used to measure the images of GA–AuNPs.

### 4.3. AuNPs Size and Zeta Potential Measurements

The particle size and zeta potential of GA–AuNPs were measured using the ELSZ-2000 zeta-potential and particle size analyzer (Otsuka Tech Electronics Co., Ltd., Tokyo, Japan).

### 4.4. Preparation of Stock Solutions of GA-AuNPs

The freeze-drying method was used to measure the weight of nanoparticles. Freeze-dried particles were evaluated to estimate the solid content, and dried-nanoparticles stock solution was prepared at 10 mg/mL concentration, then diluted to a final working concentration of 0, 1, 5, 10, 25, 50, or 100 μg/mL in MEM (minimum essential medium) medium.

### 4.5. Cell Culture

Human dermal fibroblasts CCD-966SK (Bioresources Collection and Research Center, BCRC, Hsinchu, Taiwan) were grown in MEM medium with 10% fetal bovine serum (Gibco BRL), 1.5 g/L sodium bicarbonate, 0.1 mM non-essential amino acid, 1% penicillin (100 units/mL)/streptomycin (100 μg/mL), and 1 mM sodium pyruvate in an incubator at 37 °C, humidified with 95% air and 5% CO_2_.

### 4.6. Cell Viability Assay

Cell cytotoxicity was measured using CellTiter 96^®^ aqueous one solution proliferation assay kit. CCD-966SK (1 × 10^5^ cells/well) were incubated in 96-well plates and treated with glucose or GA–AuNPs at various concentrations for 24 h at 37 °C. After incubation, 20 μL CellTiter 96^®^ solution was added to each well and incubated for 2 h at 37 °C. The absorbance of the well contents was measured at 490 nm using a Multiskan Sky Microplate Spectrophotometer (Thermo Fisher Scientific).

### 4.7. Evaluation of the Intracellular Reactive Oxygen Species (ROS) Production

H_2_DCFDA (2′,7′-dichlorodihydrofluorescein diacetate) was employed to detect the intracellular generation of ROS. The irradiated cells were collected and incubated with 10 µM H_2_DCFDA for 20 min prior to harvesting and then washed with PBS. ROS generation was analyzed by flow cytometry (Beckman FC500, Beckman Coulter, Brea, CA, USA).

### 4.8. Real-Time Polymerase Chain Reaction

RNeasy mini kit (Geneaid Biotech Ltd., Taipei, Taiwan) was used to isolate total RNA from the cells according to the manufacturer’s instructions. Reverse transcriptase reaction was carried out with 2 μg of total RNA using M-MLV reverse transcriptase (Promega) according to manufacturer’s instructions. ABI 7500 Real-Time PCR System (Applied Biosystems Inc., Foster City, CA, USA) was used to perform quantitative PCR using the GoTaq qPCR Master Mix (Promega). The following thermocycling conditions were applied: incubation at 95 °C for 2 min, 40 cycles of amplification at 95 °C for 15 s each, and then 60 °C for 60 s. The threshold cycle was determined as the cycle number of the fluorescence from the amplified PCR products. Glyceraldehyde-3-phosphate dehydrogenase (GAPDH) expression was used as the normalization of mRNA levels in each sample to define the PCR arbitrary units of each gene. Primer sequences used are listed as below: GAPDH, 5′-GAAATCCCATCACCATCTTCCAGG-3′ (forward), 5′-GAGCCCCAGCCTTCTCCATG-3′ (reverse); MMP-1, 5′-CTGGCCACAACTGCCAAA-3′ (forward), 5′-CTGTCCCTGAACAGCCCAGTACTTA-3′ (reverse); MMP-3, 5′-ATTCCATGGAGCCAGGCTTTC-3′ (forward); 5′-GCATTTGGGTCAAACTCCAACTGT-3′ (reverse).

### 4.9. Western Blot Analysis

After specific treatment, cells were lysed with RIPA lysis buffer (Millipore-Sigma, Burlington, MA, USA), and the protein concentration in the sample was measured using the PierceTM Rapid Gold BCA Protein Assay Kit (Thermo Fisher Scientific). Sodium dodecyl sulfate–polyacrylamide gel electrophoresis (SDS-PAGE) were used to fraction protein samples, which were then transferred to PVDF membranes at room temperature. Appropriate primary antibodies and horseradish peroxidase-conjugated secondary antibodies were added and individually incubated on membranes at 4 °C. This was followed by detection using Western Lightning™ Chemiluminescence Reagent Plus (PerkinElmer, Waltham, MA, USA).

### 4.10. Collagenase Activity—Zymography Assay

Cell culture supernatants were harvested after specific treatment and the inactivated zymogen forms of collagenase were activated with 1 mM p-aminophenylmercuric acetate (APMA). Collagen Degradation/Zymography assay kit (Abcam) was used to determine collagenase activity according to the manufacturer’s instructions. Collagenase activity was normalized to either total protein or total condition medium volume.

### 4.11. Collagen Zymography

The ability of cells to activate MMP-1 was determined by collagen zymography. Cells were incubated in a serum-free medium containing high-glucose or GA–AuNPs for 24 h. The culture medium was mixed with SDS-loading buffer (0.125 M Tris-HCl, pH 6.8, 4% SDS and 0.02% bromophenol blue) and incubated for 30 min at 37 °C. Samples were electrophoresed in 10% polyacrylamide gel containing 0.1% collagen. Gels were washed in 2.5% Triton X-100 for 30 min with constant mechanical rotation at room temperature. Gels were then incubated at 37 °C for 48 h in 50 mM Tris- HCl (pH 7.5) containing 150 mM NaCl and 5 mM CaCl_2_. Gels were stained with 0.1% Coomassie Blue R-250, then collagenases were identified as clear bands.

### 4.12. Statistical Analysis

All data were presented as the mean ± standard deviation (SD). Statistical analyses were performed using GraphPad Prism 9.01 (GraphPad software, La Jolla, CA, USA) with unpaired Student’s *t*-test. The results with * *p* < 0.05 were acknowledged as statistically significant. A scanning densitometer was used to quantify the results of Western blots. Changes seen in protein levels relative to β-actin-loading controls were displayed at the bottom of immunoreactive bands. Gatan Digital Micrography software was employed to analyze the particle size distribution through TEM images.

## 5. Conclusions

In conclusion, the data presented in this study suggest that ROS-dependent ASK1-p38 MAPK/ERK activation and c-Jun/c-Fos/ATF-2 phosphorylation are crucial for high glucose-mediated MMP-1 expression. High-glucose also activated NFκB through the generation of ROS, and induced MMP-1 upregulation in dermal fibroblasts. GA–AuNPs inhibited oxidative stress, the upregulation of MMP-1, and the degradation of type I collagen by high-glucose. Our data suggested that GA–AuNPs are valuable as an active ingredient in anti-aging products, particularly for high glucose-induced skin aging.

## Figures and Tables

**Table 1:** TEM-derived size distributions of gallic acid-coated gold nanoparticles with various levels of pH synthesis.

pH	Median Size (nm)	Mean Size (nm) ± SD	Particle Numbers
6	36.43	36.02 ± 5.80	*n* = 129
9	26.02	26.23 ± 4.16	*n* = 172
11	29.88	30.30 ± 3.98	*n* = 119

**Table 2:** Mean size (by DLS measurement) and zeta potential of gallic acid-coated gold nanoparticles with various levels of pH synthesis.

pH	Mean Size (nm) ± SD	Zeta Potential (mV) ± SD	Polydispersity Index
6	52.0 ± 18.1	61.30 ± 2.84	0.259
9	32 ± 20.8	48.26 ± 1.84	0.311
11	40.8 ± 15.4	–49.63 ± 2.11	0.141

## Data Availability

Data is contained within the article.

## Supplementary Materials

The following are available online at www.mdpi.com/article/10.3390/ph14111071/s1. Figure S1: TEM images for GA-AuNPs prepared at pH 6, pH 9 and pH 11.
