# Supplementary figures and images for: Evaluation of Gallic Acid-Coated Gold Nanoparticles as an Anti-Aging Ingredient
Source: Pharmaceuticals (Basel). 2021 Oct 22;14(11):1071. doi: 10.3390/ph14111071 (PMC8624563; doi:10.3390/ph14111071)

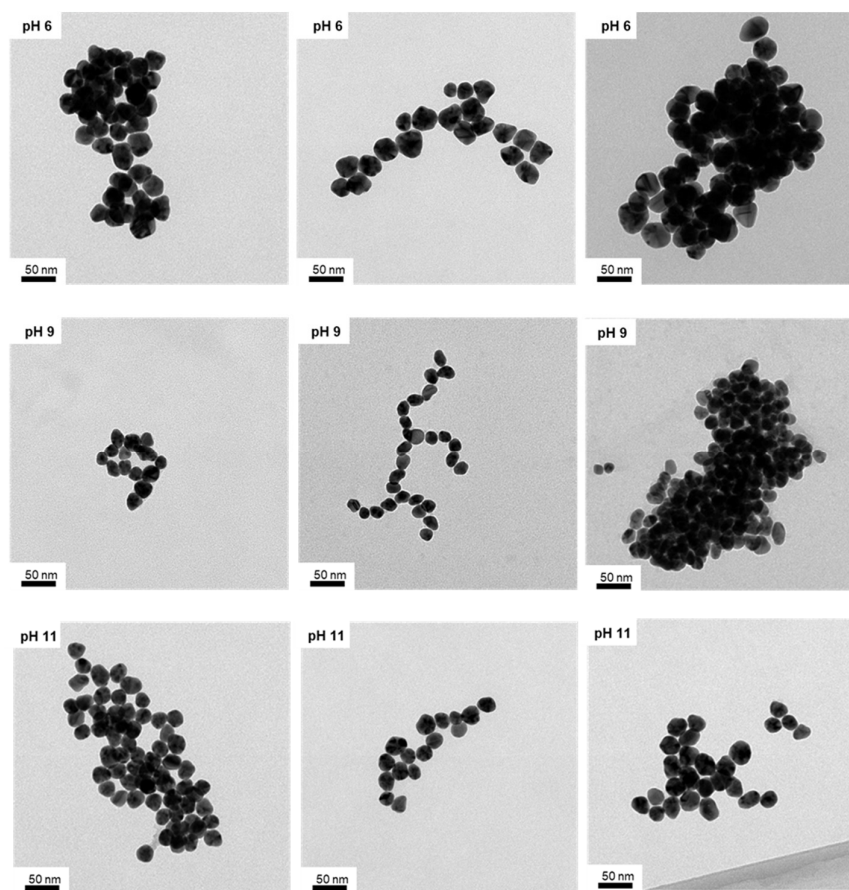

**Supplementary Figure S1.** TEM images for GA-AuNPs prepared at pH 6, pH 9 and pH 11.

Supplement: Supplementary file 1 [file pharmaceuticals-14-01071-s001.zip › pharmaceuticals-1430457-supplementary.pdf]
